# Vertical Center-of-Mass Braking and Motor Performance during Gait Initiation in Young Healthy Adults, Elderly Healthy Adults, and Patients with Parkinson’s Disease: A Comparison of Force-Plate and Markerless Motion Capture Systems

**DOI:** 10.3390/s24041302

**Published:** 2024-02-17

**Authors:** Arnaud Simonet, Arnaud Delafontaine, Paul Fourcade, Eric Yiou

**Affiliations:** 1 LADAPT Loiret, Centre de Soins de Suite et de Réadaptation, 45200 Amilly, France; simonet.arnaud@ladapt.net; 2 CIAMS, Université Paris-Saclay, 91190 Paris, France; arnaud.delafontaine@ulb.be (A.D.); paul.fourcade@universite-paris-saclay.fr (P.F.); 3 CIAMS, Université d’Orléans, 45067 Orléans, France; 4 Département de Chirurgie Orthopédique, Université Libre de Bruxelles, 1050 Bruxelles, Belgium

**Keywords:** markerless motion capture, force-plate, gait initiation, braking index, motor performance, Bland and Altman, Bayes factor 01, Parkinson’s disease, healthy adults, biomechanics

## Abstract

Background. This study tested the agreement between a markerless motion capture system and force-plate system (“gold standard”) to quantify stability control and motor performance during gait initiation. Methods. Healthy adults (young and elderly) and patients with Parkinson’s disease performed gait initiation series at spontaneous and maximal velocity on a system of two force-plates placed in series while being filmed by a markerless motion capture system. Signals from both systems were used to compute the peak of forward center-of-mass velocity (indicator of motor performance) and the braking index (indicator of stability control). Results. Descriptive statistics indicated that both systems detected between-group differences and velocity effects similarly, while a Bland–Altman plot analysis showed that mean biases of both biomechanical indicators were virtually zero in all groups and conditions. Bayes factor 01 indicated strong (braking index) and moderate (motor performance) evidence that both systems provided equivalent values. However, a trial-by-trial analysis of Bland–Altman plots revealed the possibility of differences >10% between the two systems. Conclusion. Although non-negligible differences do occur, a markerless motion capture system appears to be as efficient as a force-plate system in detecting Parkinson’s disease and velocity condition effects on the braking index and motor performance.

## 1. Introduction

Locomotion is a complex task that requires body stabilization and body propulsion simultaneously. Gait initiation, the transient period between a quiet standing posture and steady-state walking [1], is a locomotor task classically used in the literature to investigate the capacity of individuals to meet these two requirements. This capacity can be evaluated using biomechanical indicators.

A biomechanical indicator of body propulsion capacity is the peak velocity of the center of mass along the progression direction [1]. According to the laws of mechanics, this velocity reflects the total amount of propulsive forces applied to the body during the gait initiation process. As an extension, this indicator is used to quantify motor performance of gait initiation. The quantification of body propulsion capacity is important in the gait evaluation context since the goal of locomotion is to propel the body in the desired direction.

The braking index, introduced by Do and colleagues [2], is a biomechanical indicator of body stabilization capacity. In young healthy adults, these authors reported that the vertical velocity of the center of mass reaches a downward-oriented peak at around mid-execution, which had previously been ascribed to a center-of-mass fall under the gravity effect. In this population, the velocity reverses direction to reach a value close to zero at the time of swing-foot contact, indicating that the fall of the center of mass is actively braked. The central nervous system thus prepares for swing-foot contact by reducing the center of mass’s vertical velocity in order to create a smooth landing and attenuate the mechanical transmission of the impact of vertical force through the whole body. In contrast, the peak of downward-oriented velocity is often reached at the time of swing-foot contact (or close to this instant) in patients with Parkinson’s disease [3,4], thus indicating that the fall of the center of mass is not actively, but passively, braked following foot landing. The braking index reflects this capacity of the central nervous system to brake the center-of-mass fall. Alteration in this capacity is known to increase the risk of a fall.

These two velocity-based indicators have been used extensively in the literature to quantify the postural disorders that occur in physiological aging [5,6,7] and in various neurological conditions, such as Parkinson’s disease [8], stroke [9], supranuclear palsy [10], etc. For example, the decreased step length and anteroposterior velocity during gait initiation are typical characteristics of patients with Parkinson’s disease, mainly resulting from the degeneration of central dopaminergic systems. In addition, the decreased braking capacity that patients with Parkinson’s disease often encounter has been reported to contribute to their gait disorders and postural instability, perhaps as a result of nondopaminergic lesions, possibly at the mesencephalic level [3]. Recent results [4] obtained in patients with Parkinson’s disease initiating gait, further showed that anteroposterior (length and velocity) and vertical (braking capacity) gait initiation parameters are controlled by two distinct systems within the basal ganglia circuitry, representing, respectively, locomotion and balance [4].

Thus, it is clear from the literature that the analysis of gait initiation through these biomechanical indicators of stability and performance is relevant for a better understanding of the physiopathology of Parkinson’s disease. To compute these indicators, researchers currently use two systems and related methods: the “force-plate” system and the “marker-based motion capture” system. In the force-plate system method, ground reaction forces are recorded with a force-plate to compute the three-dimensional instantaneous acceleration of the center of mass (according to Newton’s second law). The three-dimensional center-of-mass velocity is then obtained through the single integration of this signal. This method has been used widely in both healthy [1,11,12,13,14,15,16,17] and pathological subjects [3,4,10,18,19], and can be considered the “gold standard” to measure the braking index and motor performance of gait initiation. This method offers at least two advantages: (i) it requires no body preparation, thus reducing the duration of the experiments, and (ii) it requires no approximation of the location of the center of mass of each body segment to compute the whole-body center-of-mass kinematics, as is the case for the marker-based motion capture. However, this method has at least three drawbacks: (i) it provides no information on body segment kinematics, (ii) it requires highly trained operators for data processing, and (iii) to record the entire gait initiation process, step length must not exceed the dimensions of the force-plate (which may be an issue when subjects initiate gait at maximal velocity on a single, small force-plate).

The marker-based motion capture typically tracks reflective markers that are attached to the skin (or clothing) using the infrared cameras of a motion capture system to estimate the three-dimensional motion of body segments. A conventional marker-based motion capture requires thirty-eight markers to compute the whole-body center-of-mass position [20]. In the marker-based motion capture, this positional signal is then derived to obtain the three-dimensional whole-body center-of-mass velocity. One advantage of the marker-based motion capture is that it can provide information on both whole-body center-of-mass and body segment kinematics during gait initiation [13,21]. However, the marker-based motion capture does have several significant drawbacks. It requires extensive experimental set-up, particularly due to the large number of markers required, which can be a serious issue in experiments involving frail participants who may quickly become tired and/or bored. The marker-based motion capture also requires a controlled environment [22,23] that may alter participants’ natural movements, due to their awareness of being observed [24]. In addition, the literature stresses that the potential misplacement of markers and the skin motion relative to bones may lead to inaccuracies on whole-body center-of-mass kinematics [25,26,27,28]. Finally, like the force-plate system, the marker-based motion capture system is expensive, requires time-intensive data processing that can introduce errors [22,23,24,29,30,31], and requires highly trained operators for data processing.

So, there is currently a need to develop more accessible systems that provide researchers and clinicians reliable values of whole-body kinematics to compute the braking index and motor performance of gait initiation. The markerless motion capture system is recent, innovative technology that has shown potential in overcoming the limitations of the force-plate system and marker-based motion capture mentioned above. A markerless motion capture system uses standard video and deep learning-based software instead of infrared cameras to detect body segment landmarks directly from digital images [32,33,34,35,36]. Thus, it eliminates many of the manual processing steps and sources of error inherent to the marker-based motion capture. Recent studies have investigated the reliability of the markerless motion capture system in quantifying three-dimensional lower-limb joint kinematics and/or kinetics during various locomotor tasks (e.g., [35,37,38,39,40]). These studies typically compared data obtained from a markerless motion capture system vs. marker-based motion capture in young healthy adults, with sometimes contrasting results. For example, in Kanko et al.’s work [35], participants performed sessions of over-ground walking trials separated by an average of 8.5 days. Three-dimensional pose estimations from a markerless motion capture system were used to compute lower-limb joint angles. These authors showed that the gait kinematics data provided by this system were as reliable as those from the marker-based motion capture, as assessed with inter-session variability, inter-trial variability, and the variability ratio between sessions. Kanko et al. [39] further showed that these two systems provided an average distance root mean square between corresponding joint centers of less than 2.5 cm for all joints except the hip, which was 3.6 cm. Lower-limb segment angles indicated that the segment pose estimations from both systems were very similar, with a distance root mean square of less than 5.5° for all segment angles except those representing rotations about the long axis of the segment. Ito et al. [37] added that, during walking, squatting, and forward hopping, sagittal plane angles were most comparable between the marker-based motion capture and the markerless motion capture system at the knee joint followed by the ankle and hip, while frontal and transverse plane angles were not. Song et al. [40] concurrently captured lower-limb kinematics in participants performing eight daily living and exercise movements. These authors found that the estimates from the markerless motion capture system were very similar to the marker-based motion capture in the ankle and knee joint angles and moments. There were more differences between the two systems for hip angles and moments, especially during rapid movements such as running. Finally, Tang et al. [38] reported that, during walking on a treadmill, the markerless motion capture system provided higher values than the marker-based motion capture for peak hip extension and flexion moments, but, in contrast to Song et al. [40], it also provided higher values for the knee flexion moment and ankle plantarflexion moment (along with higher joint powers).

In addition to these disparate results concerning the reliability of the markerless motion capture system, it is worth noting that these studies systematically used the marker-based motion capture as the gold standard for these measures of the different body part movements. Also, only the kinematics (and/or the kinetics) of lower-limb joints in young healthy adults were considered. Therefore, the question of whether a markerless motion capture system is a reliable technique for computing whole-body center-of-mass kinematics during gait initiation—and the associated braking index and motor performance—in both young healthy adults and patients with postural disorders remains to be clarified.

So, this study tested the agreement between the markerless motion capture system and the force-plate system (considered here as the gold standard) to estimate the braking index and motor performance during gait initiation in healthy adults (both young and elderly) and in patients with Parkinson’s disease. This agreement was investigated using the following statistical methods: the Bland–Altman method (classically used to compare two different measurement techniques [41]), classical descriptive statistics, and Bayes factor 01. Bayes factor 01 is a ratio between two competing statistical models represented by their evidence. It is used to quantify support for one model over the other [42]. In this study, it was used to contrast the two following hypotheses: H0 (the “null hypothesis”, i.e., both systems provide the same braking index and motor performance) vs. H1 (the “alternative hypothesis”, i.e., both systems provide a different braking index and motor performance). Note that with Bayes factor 01, evidence can be quantified in favor of or against a null hypothesis, which cannot be carried out using the *p*-value provided by classical descriptive statistics.

## 2. Materials and Methods

### 2.1. Participants

Three groups of participants (n = 33) were involved in this experiment: young healthy adults, healthy elderly adults, and patients with Parkinson’s disease (cf. Table 1 for the anthropometrical features and evaluation scores of the patients with Parkinson’s disease). The inclusion criteria were as follows: aged between 20 and 30 years for the young healthy adults, and aged between 60 and 80 years for the healthy elderly adults and patients with Parkinson’s disease. The exclusion criteria were as follows: walking with aids; visual, hearing, or orthopedic problems; identified neurological disorders (other than Parkinson’s); dementia; cognitive impairments (i.e., a score < 25 on the Mini Mental State Exam and Montreal Cognitive Assessment); and a medical history of falling. All of the participants gave their written consent after having been informed of the nature and purpose of the experiment. The study was conducted in accordance with the Declaration of Helsinki, and approved by the “Comité de Protection des Personnes Ile-de-France XI” under identification number 19028-60429 (date of approval: 6 November 2019). 

### 2.2. Experimental Set-Up, Tasks, and Conditions

**Figure 1:**
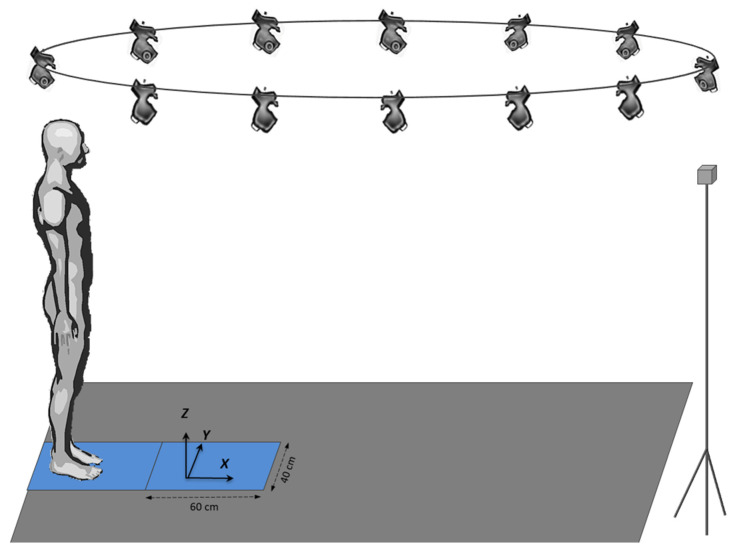
Experimental set-up showing the markerless motion capture system, the force-plate system, and the walking track.

The experiments were conducted in the Biomechanics laboratory of the LADAPT Loiret rehabilitation center (Amilly, France). The physical conditions (room temperature and time of day) were the same for all participants. The participants initially stood barefoot on a force-plate with a second force-plate positioned in front of it (see Figure 1). This system of two force-plates in series allowed biomechanical recordings of the entire gait initiation process. Both force-plates (0.4 × 0.60 m, AMTI, Watertown, MA, USA) were embedded at the beginning of a six-meter-long walking track (Figure 1). The capacity of the two force-plates was 9000 N for the vertical force, 4450 N for the anteroposterior force, and 4450 N for the mediolateral force. In the present study, only the anteroposterior and vertical forces were analyzed. Force-plates had a maximum capture rate of 400 Hz.

A markerless motion capture system equipped with 12 hybrid miqus cameras (Qualisys, Göteborg, Sweden) with a 2 million resolution and a capture rate of 85 images per second in a full high-definition mode was synchronized with both force-plates with Qualisys Track Manager software (version 2021.2, build 6720). The cameras were distributed all along the walking track with an approximate 2 m distance between them, and were connected in series with an RJ45 cable and power supply. This system recorded the participants’ complete motion on the walking track. The data obtained by the cameras were then transferred to Theia software (version 2021.2.0.1675, Theia3D, Kingston, ON, Canada), which reconstructed body kinematics. The signals from these two systems were used to compute the participants’ whole-body center-of-mass kinematics (cf. “Raw Data Processing”).

In the initial standing posture, the feet were positioned shoulder-width apart, the arms rested alongside the trunk, and the gaze was directed forward to a small target at eye level (2 cm diameter, 6 m away). The locations of the heel and big toe of each foot were marked on the first force-plate with strips of adhesive tape and were used as a visual reference on which the participants positioned themselves after each trial under the supervision of the experimenters. From this initial posture, the participants performed series of gait initiation in a “spontaneous velocity condition” (n = 5 trials) and in a “maximal velocity condition” (n = 5 trials).

In both velocity conditions, the participants were instructed to initiate gait under their own initiative following an auditory signal delivered by the experimenter, and then continued walking straight ahead until they reached the end of the track. It was made clear to them that they had to initiate gait only when they felt ready following the auditory signal, i.e., that it was not a reaction time situation. They were also asked to stand immobile in the initial standing posture while waiting for the starting signal. These instructions were repeatedly recalled to participants in the course of the experiment.

One practice trial was conducted in each velocity condition (not recorded) to ensure that the participants clearly understood the instructions and that the material was operational. A 10 s rest time between trials, and a 2 min rest time between velocity conditions were imposed to avoid the effects of fatigue. Both conditions of velocity were randomized across participants to avoid any rank effects. The visualization of the task and the experimental force-plate set-up can be found in Simonet et al.’s work [15].

### 2.3. Raw Data Processing

*Force-plate system.* The force-plate system recorded the three-dimensional components of the ground reaction force and moment vector acting at the surface of each force-plate. The instantaneous three-dimensional components of the ground reaction force vector recorded by each force-plate were algebraically summed to obtain the three-dimensional components of the “global” ground reaction force vector, i.e., the ground reaction force acting on the whole-body center of mass. The instantaneous accelerations of the center of mass along the anteroposterior and vertical direction were computed from this vector according to Newton’s second law [15]. To obtain the vertical acceleration of the center of mass, the participant’s weight was subtracted from the vertical component of this ground reaction force vector. The instantaneous center-of-mass velocity along the anteroposterior and vertical direction was computed by the single numerical integration of the center-of-mass acceleration using the rectangles method and considering the initial center-of-mass velocity as null.

*Markerless motion capture system.* The markerless motion capture system recorded the full-body three-dimensional kinematics. The instantaneous center-of-mass (COM) velocity along the vertical direction (z’COM(t)) was computed as follows:

z’COM(t) = [zCOM(t) − zCOM(t_−1_)] ∗ F

where zCOM(t) and zCOM(t_−1_) are the vertical position of the center of mass at time t and at the previous frame (t_−1_), respectively; F is the acquisition frequency.

Similarly, the instantaneous center-of-mass velocity along the anteroposterior direction (x’COM(t)) was computed as follows:

x’COM(t) = [xCOM(t) − xCOM(t_−1_)] ∗ F

where xCOM(t) and xCOM(t_−1_) are the anteroposterior position of the center of mass at time t and at the previous frame (t_−1_), respectively; F is the acquisition frequency.

The acquisition frequency was 85 Hz for both the markerless motion capture system and the force-plate system. This frequency corresponds to the limit of the markerless motion capture system in a full high-definition mode. The same frequency was used for the force-plate system to allow comparison with the markerless motion capture system. For both systems, data were filtered with a no-lag low-pass Butterworth order filter with a 15 Hz cut-off frequency [15]. Qualisys Track Manager software was used to synchronize the signals from both systems.

### 2.4. Experimental Variables

**Figure 2:**
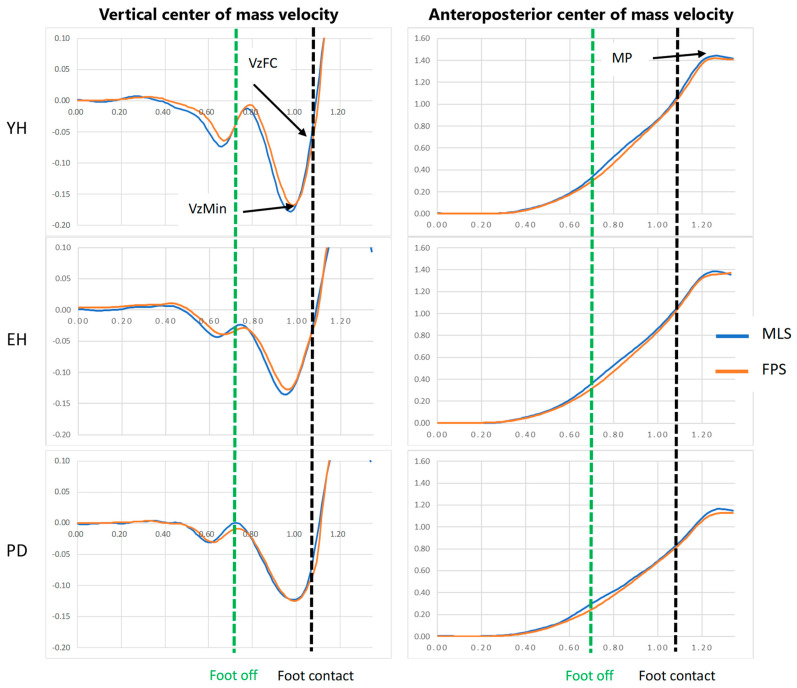
Typical biomechanical traces of the center-of-mass velocity along the vertical and anteroposterior directions obtained with the force-plate system (FPS, red trace) and markerless motion capture system (MLS, blue trace) in the three groups. Reported are the mean traces of the five trials obtained in the maximal velocity condition in a representative participant for the young healthy adults (YH), healthy elderly adults (EH), and patients with Parkinson’s disease (PD). Velocity (ordinate) is expressed in meters/second. Time (abscissa) is expressed in seconds. MP, Vzmin, and VzFC: motor performance, peak negative velocity, and vertical center-of-mass velocity at the time of foot contact, respectively. Note the good agreement between both systems in each plot.

*Braking index*. The braking index (BI) reflects the central nervous system’s ability to actively brake the vertical fall of the center of mass under gravity [3,4,18,19]. It is a classical indicator of stability control that is computed as follows:

BI = [Vzmin − VzFC]/Vzmin

where Vzmin and VzFC are the peak of downward center-of-mass vertical velocity and vertical center-of-mass velocity at foot contact, respectively (Figure 2).

*Motor performance*. Motor performance corresponds to the peak of the anteroposterior center-of-mass velocity (Figure 2). The braking index and motor performance were computed with the force-plate system and markerless motion capture system.

### 2.5. Statistics

To investigate the agreement between the force-plate system and the markerless motion capture system, a Bland–Altman analysis was conducted on a trial-by-trial basis within each group (i.e., patients with Parkinson’s disease, young healthy adults, and healthy elderly adults) and the experimental condition of velocity (i.e., spontaneous velocity, maximal velocity). The values provided by the force-plate system were considered as the gold standard against which the values provided by the markerless motion capture system were compared. Bland–Altman plots were generated, with the horizontal axis representing the average of the braking index values (or motor performance) obtained with the force-plate system and the markerless motion capture system, and the vertical axis representing the difference between the two methods. The accuracy of the markerless motion capture system was estimated with bias, corresponding to the mean difference (d) and the standard deviation of the differences. The normality of the differences was checked using the Shapiro–Wilk test. The reliability of the markerless motion capture system was estimated with the dispersion of the differences within each plot, as quantified with the 95% limits of agreement. These limits corresponded to d + 1.96 standard deviation (upper limit) and d − 1.96 standard deviation (lower limit). Absolute and relative values of the biases and agreement limits were reported.

The Bland–Altman analysis was completed by descriptive statistics, which included means and standard deviations of the braking index, motor performance, and corresponding biases. Repeated measures ANOVAs with the velocity (two levels: spontaneous vs. maximal) and the system (two levels: markerless motion capture system vs. force-plate system) as within-subject factors and the group (three levels: young healthy adults vs. healthy elderly adults vs. patients with Parkinson’s disease) as the between-subject factor were conducted on each variable. Tukey post hoc tests were used when necessary. The alpha level was set at 0.05.

Finally, Bayes factor 01 was computed for each variable to contrast the following hypotheses: H0 (the null hypothesis, i.e., “both systems provide the same braking index and motor performance”) vs. H1 (the alternative hypothesis, i.e., “both systems provide a different braking index and motor performance”). It is generally admitted that if Bayes factor 01 is above 3, then the null hypothesis is evident [43,44,45].

## 3. Results

### 3.1. Description of the Biomechanical Traces

A visual analysis of the traces reported in Figure 2 shows that the global time courses of the velocity of anteroposterior and vertical center of masses obtained with the force-plate system and the markerless motion capture system were very similar. They were also very similar across the three groups (young healthy, healthy elderly, patients with Parkinson’s disease) and the two velocity conditions (only the maximal velocity condition is shown in Figure 2). More specifically, the velocity of the vertical center of mass reached two successive downward-oriented peaks, the first approximately a few milliseconds before the swing-foot was off the ground, and the second (larger) before swing-foot contact. The direction of the velocity trace then reversed, showing that the fall of the center of mass was actively braked before foot contact. The anteroposterior center-of-mass velocity increased to reach peak velocity a few milliseconds after swing-foot contact.

### 3.2. Bland–Altman Analysis

**Figure 3:**
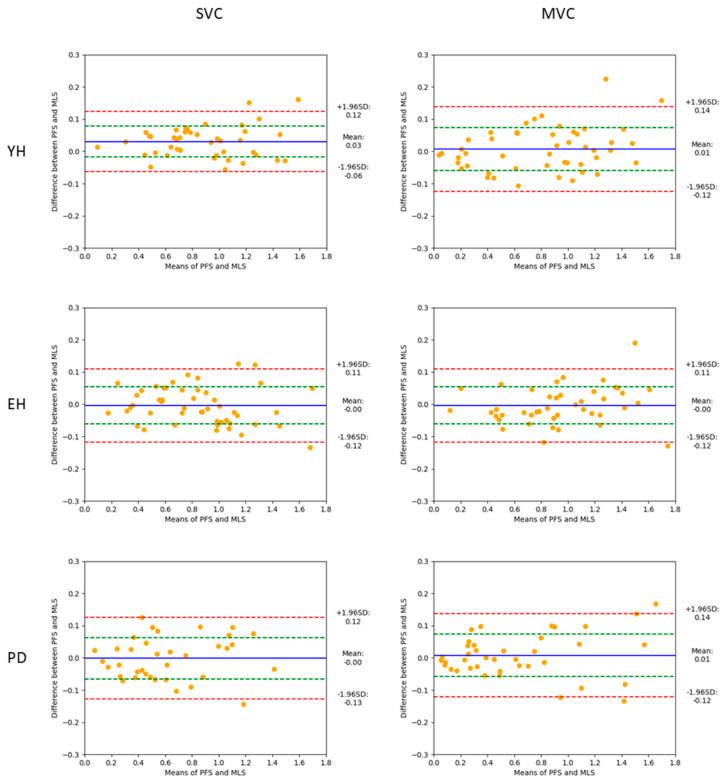
Bland–Altman plots showing the braking index values obtained with the two measurement systems (mean values, abscissa), against the difference between these two systems (ordinate), for the young healthy adults (YH), healthy elderly adults (EH), and patients with Parkinson’s disease (PD). Each point represents one trial with one participant. Reported are the absolute values obtained in the spontaneous (SVC) and maximal (MVC) velocity conditions. FPS and MLS: force-plate system and markerless motion capture system, respectively. For each plot, the 95% limits of agreement (red dotted lines), standard deviation (green dotted line), and bias (blue full line) are reported.

*Braking index*. In the spontaneous velocity condition (Figure 3, left), the Bland–Altman analysis showed that 95% of the absolute differences between the two systems ranged between −0.06 and 0.12 m/s for the young healthy adults (which corresponded to a range of relative differences of between −7 and 14%), −0.12 and 0.11 m/s for the healthy elderly adults (−17 and 16%), and −0.13 and 0.12 m/s for the patients with Parkinson’s disease (−28 and 24%). In the maximal velocity condition (Figure 3, right), the Bland–Altman analysis showed that 95% of the absolute differences between the two systems ranged between −0.12 and 0.14 m/s for the young healthy adults (−25 and 20%), −0.12 and 0.11 m/s for the healthy elderly adults (−16 and 14%), and −0.12 and 0.14 m/s for the patients with Parkinson’s disease (−27 and 25%). Therefore, the limits of relative agreement were greater for the patients with Parkinson’s disease than for the two healthy groups. The absolute and relative biases were virtually zero in each velocity condition and group.

**Figure 4:**
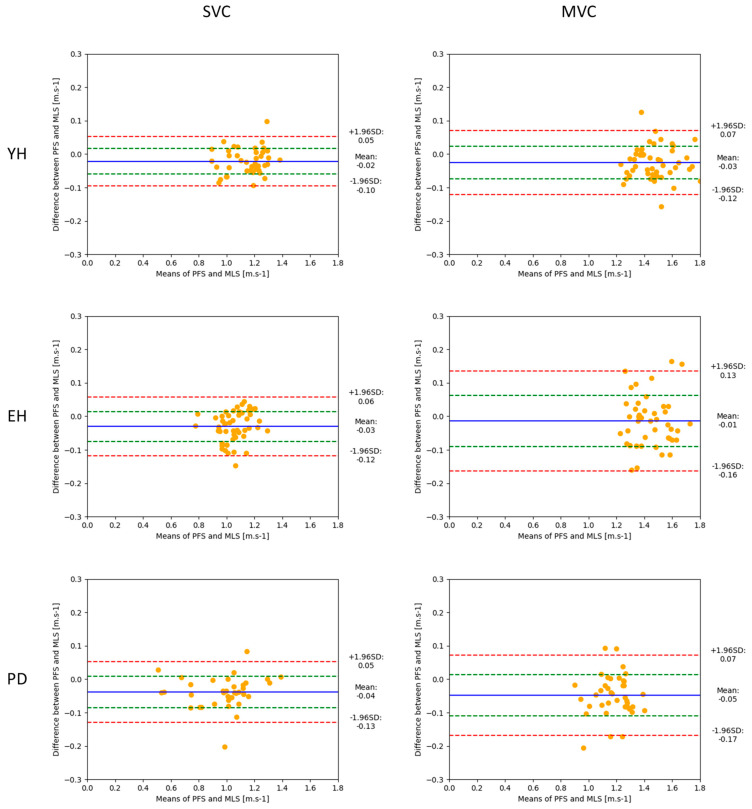
Bland–Altman plots showing motor performance (peak of anteroposterior center-of-mass velocity) obtained with the two measurement systems (mean values, abscissa), against the difference between these two systems (ordinate), for the young healthy adults (YH), healthy elderly adults (EH), and patients with Parkinson’s disease (PD). Each point represents one trial with one participant. Reported are the absolute values obtained in the spontaneous (SVC) and maximal (MVC) velocity conditions. FPS and MLS: force-plate system and markerless motion capture system, respectively. For each plot, the 95% limits of agreement (red dotted lines), standard deviation (green dotted line), and bias (blue full line) are reported.

Motor performance. In the spontaneous velocity condition (Figure 4, left), the Bland–Altman analysis showed that 95% of the absolute differences between the two systems ranged between −0.10 and 0.05 m/s for the healthy young adults (which corresponded to a range of relative differences between −9 and 5%), −0.12 and 0.06 m/s for the healthy elderly adults (−12 and 6%), and −0.13 and 0.05 m/s for the patients with Parkinson’s disease (−15 and 6%). In the maximal velocity condition (Figure 4, right), the Bland–Altman analysis showed that 95% of the absolute differences between the two systems ranged between −0.12 and 0.07 m/s for the young healthy adults (−8 and 5%), −0.16 and 0.13 m/s for the healthy elderly adults (−12 and 9%), and −0.17 and 0.07 m/s for the patients with Parkinson’s disease (−16 and 7%). The absolute and relative biases were virtually zero in each velocity condition and group.

### 3.3. Descriptive Statistics

**Figure 5:**
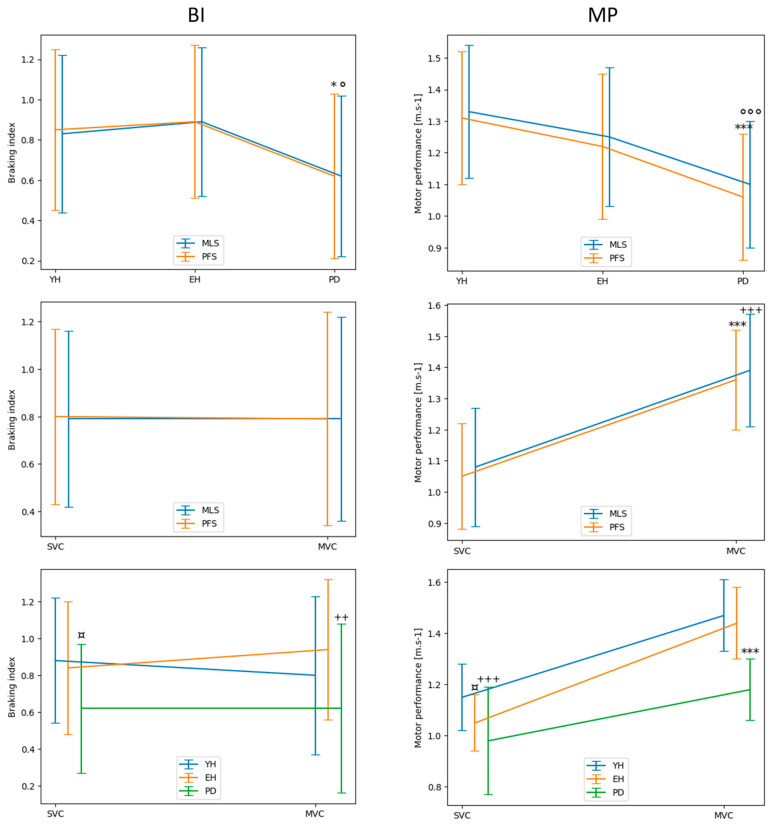
Descriptive statistics comparing the braking index (left panels) and motor performance (right panels) between the systems, groups, and velocity conditions. Reported are mean values (all participants together) ±1 standard deviation. Upper plots. * and ***: significant difference between the patients with Parkinson’s disease (PD) and both the healthy young (YH) and elderly (EH) adults with *p* < 0.05 and *p* < 0.001, respectively, as detected by the force-plate system (FPS). ° and °°°: significant difference between the patients with PD and both the YH and EH adults with *p* < 0.05 and *p* < 0.001, respectively, as detected by the markerless motion capture system (MLS). Middle plots. *** and +++: significant difference between spontaneous velocity condition (SVC) and the maximal velocity condition (MVC), as detected by the FPS and the MLS, respectively, with *p* < 0. 001. Lower plots. ¤ and ++: significant difference between the patients with PD and both the YH and EH adults in the SVC and the MVC, respectively (with *p* < 0.05 and *p* < 0.01). ¤, +++, ***: significant difference between the YH and the EH in the SVC (with *p* < 0.05), significant difference between the YH and the patients with PD in the SVC (with *p* < 0.001), significant difference between the patients with PD and both the EH and the YH in the MVC (with *p* < 0.001). Note the superposition of the traces obtained with the two systems in both upper panels.

*Braking index*. Repeated-measures ANOVAs showed that there was a significant main effect of the group (F_2,104_ = 9.8, *p* < 0.001), with no significant main effect of the system or the velocity on the braking index. Post hoc testing further showed that the braking index was significantly higher in both the young healthy adults and the healthy elderly adults than in the patients with Parkinson’s disease in both the spontaneous velocity and the maximal velocity condition. It also showed that this between-group difference was detected similarly by both systems (cf. Figure 5 for details on the post hoc tests). There was no significant velocity X group, system X group, system X velocity, or system X velocity X group interaction.

*Motor performance*. Repeated-measures ANOVAs showed that there was no significant main effect of the system, but there was a significant main effect of the group (F_2,104_ = 51.3, *p* < 0.001) and the velocity (F_1,104_ = 250.2, *p* < 0.001) on motor performance. Post hoc testing further showed that performance decreased significantly between the young healthy adults and the patients with Parkinson’s disease and, as expected, it was higher in the maximal velocity condition than in the spontaneous velocity condition (cf. Figure 5 for details on post hoc testing). There was also a significant group X velocity interaction on this variable (F_2,104_ = 6.8, *p* < 0.001). Post hoc testing showed that this interaction could be ascribed to the results that motor performance was not significantly different between the healthy elderly adults and the patients with Parkinson’s disease in the spontaneous velocity condition, while it was significantly higher in both the young healthy adults and the healthy elderly adults than in the patients with Parkinson’s disease in the maximal velocity condition (Figure 5). There was no significant system X group, system X velocity, or system X velocity X group interaction on this variable. Globally taken, these results show that both systems detected the same between-group and velocity differences in motor performance. Globally taken, these results show that the two systems detected the same between-group and velocity differences in motor performance.

### 3.4. Bayes Factor 01

**Figure 6:**
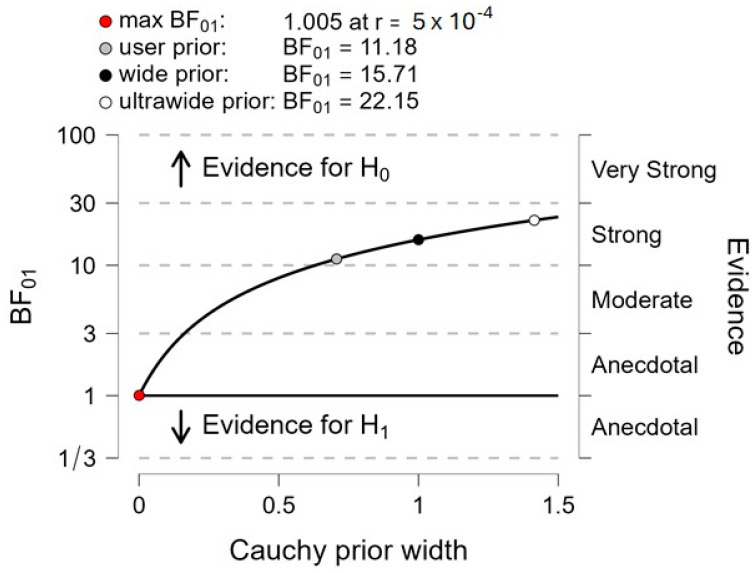
Between-system comparison with Bayes factor 01 applied to the braking index. The plot shows the Cauchy prior width (abscissa) vs. Bayes factor value (ordinate). Reported in the plot are the user value (gray dot), wide value (black dot), and ultrawide value (white dot). H0: null hypothesis (“there is no difference between both systems”); H1: alternative hypothesis (“there is a difference between both systems”).

*Braking index*. The computation of Bayes factor 01 (Figure 6) shows that the null hypothesis (H0 = “there is no difference of braking index between both systems”) was 11.18 times as likely as the alternative hypothesis (H1 = “there is a difference of braking index between both systems”), corresponding to “strong evidence”.

**Figure 7:**
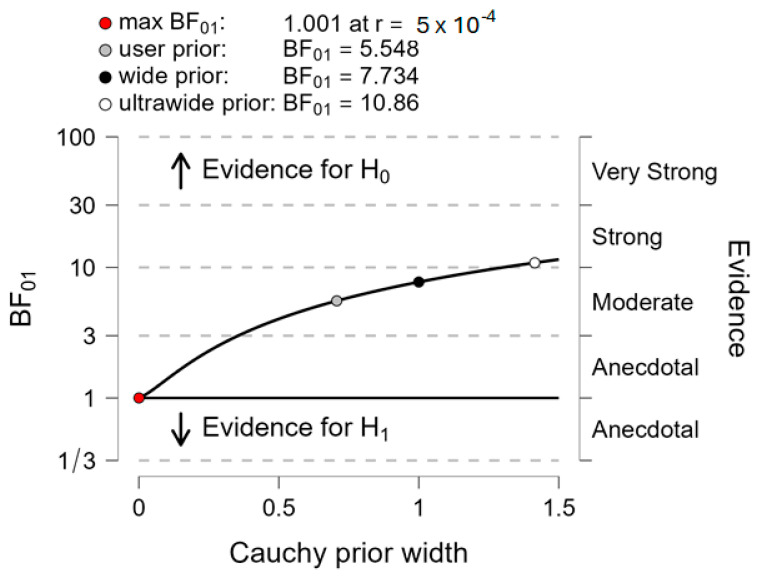
Between-system comparison with Bayes factor 01 applied to motor performance. Plots show the Cauchy prior width (abscissa) vs. Bayes factor value (ordinate). Reported in the plot are the user value (gray dot), wide value (black dot), and ultrawide value (white dot). H0: null hypothesis (“there is no difference between both systems”); H1: alternative hypothesis (“there is a difference between both systems”).

*Motor performance*. The computation of Bayes factor 01 applied to motor performance (Figure 7) shows that the null hypothesis (H0 = “there is no difference of motor performance between both systems”) was 5.55 times as likely as the alternative hypothesis (H1 = “there is a difference of motor performance between both systems”), corresponding to “moderate evidence”.

## 4. Discussion

This study tested agreement between the markerless motion capture system and the force-plate system (considered as the gold standard) to estimate the braking index and motor performance during gait initiation in healthy adults (both young and elderly) and in patients with Parkinson’s disease. Agreement was investigated using the Bland–Altman method and classical descriptive statistics, and was further tested by Bayes factor 01.

A visual analysis of Figure 2 (mean of five gait initiation trials obtained in a representative participant of each group in the maximal velocity condition) showed that there was a good superimposition of the center-of-mass velocity time-course traces when computed with the markerless motion capture system and the force-plate system, thus suggesting the existence of good agreement between the two measurement systems. Similar visual agreement between the time-course traces was also observed in the three groups when gait was initiated at a spontaneous velocity (not shown in Figure 2). The results of the different statistical methods used in this study to compare both measurement systems are in line with these visual observations. Descriptive statistics indicated that there was no significant main effect of the system on either the braking index or on motor performance. In addition, the Bland–Altman analysis showed that the mean biases of both variables were virtually zero in the spontaneous and maximal velocity conditions and for all groups, indicating that the accuracy of the markerless motion capture system was good. The computation of Bayes factor 01 further showed that there was strong evidence for the braking index and moderate evidence for motor performance that both systems provided the same values.

However, the results regarding the relative 95% limits of agreement, which reflect the dispersion of the differences between the two systems, attenuate the statement that the markerless motion capture system may be used in place of the force-plate system to measure the braking index and motor performance for all groups and velocity conditions. It can be assumed that these relative limits should be lower than an arbitrary upper 10% value (in absolute value) to validate the reliability of the alternative system (e.g., [46]), i.e., the markerless motion capture system in this experiment. Considering the braking index, the results showed that the upper relative limit (in absolute value) reached 14%, 17%, and 28% in the healthy young adults, healthy elderly adults, and patients with Parkinson’s disease, respectively, when gait was initiated at a spontaneous velocity, and 25%, 16%, and 27% in the healthy young adults, healthy elderly adults, and patients with Parkinson’s disease, respectively, when gait was initiated at maximal velocity (app. m/s). Thus, the 10% threshold was systematically exceeded, especially in the patients with Parkinson’s disease, which calls into question the reliability of the markerless motion capture system to measure the braking index.

Considering motor performance, the results showed that the upper relative limit reached 9%, 12%, and 15% in the healthy young adults, healthy elderly adults, and patients with Parkinson’s disease, respectively, when gait was initiated at a spontaneous velocity, and 8%, 12%, and 16% in the healthy young adults, healthy elderly adults, and patients with Parkinson’s disease, respectively, when gait was initiated at maximal velocity. Therefore, measurement of motor performance by the markerless motion capture system was reliable for the healthy young adults in both velocity conditions. In contrast, the upper relative limit slightly exceeded the 10% threshold for the healthy elderly adults and patients with Parkinson’s disease, which calls into question the reliability of the markerless motion capture system to measure motor performance. The better reliability on motor performance as compared to reliability on the braking index likely arises from the different expressions of these two variables, involving either two measurements (the peak of downward center-of-mass velocity and the vertical center-of-mass velocity at foot contact, for the braking index), or a single measure (the anteroposterior peak of the center of mass for motor performance), and therefore as many biases.

We should emphasize that the descriptive statistics further revealed a main effect of the group on the braking index, with no significant main effect of the system or velocity on this variable. In other words, both systems detected the same group effect, with post hoc tests revealing a higher value in both healthy young adults and healthy elderly adults than in the patients with Parkinson‘s disease, in both the spontaneous and maximal velocity condition. This altered capacity to brake the center-of-mass fall in patients with Parkinson’s disease has been repeatedly reported in the literature with a force-plate system [3,4] and is thought to reflect a deficit in postural stability control [21,47,48,49]. Thus, the results of this experiment show that the markerless motion capture system is as efficient as the force-plate system in revealing this deficit in patients with Parkinson’s disease. The descriptive statistics also revealed no significant main effect of the system, but a significant main effect of the group and velocity on motor performance. More specifically, both systems similarly detected that motor performance decreased from the young healthy adults to the patients with Parkinson’s disease and increased from the spontaneous to the maximal velocity condition. Both results are consistent with the literature [1,3,4]. Globally taken, these results show that both systems detected the same between-group difference and velocity effects for the braking index and performance. However, caution should be taken on the generality of the present results obtained in the patients with Parkinson’s disease. None of them indeed had a “freezing” behavior during any gait initiation trials. It is therefore not excluded that if they had, a larger (or a smaller) difference between the two measurement systems might have been detected for one or both variables. It is not known whether the markerless motion capture system would then be more (or less) sensitive than the force-plate system to detect a between-group difference and velocity effects for the braking index and performance.

It is important to stress that the cost of a markerless motion capture system (around EUR 120 k for the system of 12 cameras used in the present study) is much higher than the cost of the force-plate system used in the present study (around EUR 15k per force-plate). The choice to use (buy) a markerless motion capture system rather than a force-plate system might be influenced by the advantages of the former system compared to the second: (1) it provides information on body segment kinematics, (2) it does not require highly trained operators for data processing, (3) data processing is much quicker, (4) it allows the recordings of the gait initiation process and the following steps over several meters, (5) it can easily be moved outdoor. Now, in addition to its higher cost, disadvantages of this system compared to the force-plate system are the following: (i) it does not provide information on the center of pressure, and (ii) its acquisition frequency is limited to 85 Hz in a full high-definition mode. This relatively low maximal frequency might be an issue for the analysis of very rapid movements. The choice to use (buy) one system rather than the other therefore depends on the movement to be investigated and the associated biomechanical parameters to be computed, and on the budget available for the research.

Finally, it should be stressed that, besides these expensive systems, many different types of wearable low-cost sensors have already been used in the literature to analyze locomotor activities. Among them, inertial measurement units have been shown to provide a relatively reliable kinematic measure of the body part where the sensor is placed, in terms of the acceleration and angular rate of lower limbs, waist, chest, arm, etc. [50,51,52,53]. Inertial measurement units are small, easy-to-use sensors, and have a much less expensive cost (typically EUR 1k) than both a markerless motion capture system and force-plate system. However, it is not known to date whether such a device is able to provide a reliable measure of the whole-body center-of-mass kinematics during locomotor activities. A fortiori, it is not known whether it can be used to accurately compute the biomechanical parameters of the present study. An inertial measurement unit indeed provides information relative to the kinematics of a specific anatomical point. However, during locomotion, the location of the center of mass continuously moves on the body because of the movements of its different parts. Consequently, the kinematics of a single anatomical point (e.g., the navel) might not be fully representative of the center-of-mass kinematics. Current research in our laboratory is being undertaken to find the anatomical point where the inertial measurement unit should be placed by the experimenter to provide the best (or the least bad) measure of the center-of-mass kinematics during gait initiation.

## 5. Conclusions

In conclusion, the results of this study suggest that, although non-negligible differences in the braking index and motor performance do occur between the two systems, the markerless motion capture system is as efficient as the force-plate system in detecting an effect of Parkinson’s disease and velocity on the braking index and motor performance. These results add to the current literature by showing that the markerless motion capture system is as reliable as the marker-based motion capture in computing lower-limb kinematics during various locomotor activities, such as walking, squatting, forward hopping [35,37,38,39,40], etc. These results might be very useful for studies investigating postural control during gait initiation in a research or clinical setting.

## Figures and Tables

**Table 1:** Participant anthropometrical features and evaluation scores of patients with Parkinson’s disease. BMI: Body Mass Index; UPDRS-III: Unified Parkinson’s Disease Rating Scale (motor part); MoCA: Montreal Cognitive Assessment; MMS: Mini Mental State Exam. There was no significant effect of the group on the body mass, height, shoe size, and BMI.

Variable	Patients with Parkinson’s Disease (n = 12)	Healthy Young Adults (n = 10)	Healthy Elderly Adults (n = 11)
Age (years)	68.4 ± 5.1	24.7 ± 0.7	66.5 ± 3.6
Gender (female/male)	1/10	4/5	8/3
Body mass (kg)	70.5 ± 11.8	70.2 ± 13.5	63.9 ± 10.1
Height (m)	1.70 ± 0.07	1.70 ± 0.12	1.65 ± 0.06
BMI (kg/m^2^)	24.5 ± 3.6	24.7 ± 0.7	23.4 ± 3.4
Shoe size (EU)	41.3 ± 1.5	40.3 ± 3.7	39.3 ± 1.8
Time of diagnosis (years)	7.4 ± 2.8		
Hoehn and Yahr scale (points)	2.0 ± 0.6		
UPDRS-III (score)	33.8 ± 11.7		
MoCA (score)	25.7 ± 2.3		
MMS (score)	27.0 ± 1.7		

## Data Availability

Data can be made available upon request to the first author of this study.
